# Early Implanon discontinuation rate and its associated factors in health institutions of Mekelle City, Tigray, Ethiopia 2016/17

**DOI:** 10.1186/s13104-018-3992-3

**Published:** 2019-01-07

**Authors:** Tsirity G/Medhin, Kahsu Gebrekirstos Gebrekidan, Mekuria Kassa Nerea, Hagos Gerezgiher, Mebrahtom Haftu

**Affiliations:** 1 Midwifery Department, Dr. Tewelde College of Health Science, Mekelle, Tigray Ethiopia; 2 Nursing School, College of Health Science, Mekelle University, Mekelle, Ethiopia; 3 Anesthesia Department, Mekelle University, Mekelle, Ethiopia

**Keywords:** Implanon, Early discontinuation rate, Associated factors

## Abstract

**Objective:**

Contraceptive discontinuation rate is very common in most developing countries, especially removal in the first year of use is common (18–63%), and the majority of these discontinuations are among women who are still in need of contraception. So the objective of this study was assessing early Implanon discontinuation rate and its associated factors in the study area. Institutional based cross sectional study was conducted and systematic random sampling technique was employed to interview the study participants. A binary logistic regression model was used to test association.

**Result:**

In this study early Implanon discontinuation rate was 38%, 95% CI (32%, 44%). women who attend secondary [AOR: 95% CI 0.35 (0.14, 0.82)] and more than secondary school [AOR: 95% CI 0.23 (0.09, 0.59)] were less likely to remove Implanon early as compared to those illiterate. Mothers who were not counseled [AOR: 95% CI 2.45 (1.05, 5.69)] and those mothers who had a side effect of the method [AOR: 95% CI 2.66 (1.23, 5.72)] discontinue the method early. The study revealed that early Implanon discontinuation rate was high. Women’s educational level, presence of side effect and effective counseling were independent predictors of early Implanon removal.

## Introduction

Family planning contributes an important role towards, advancing maternal and newborn health, reduced maternal mortality and universal access to reproductive health care [1, 2]. Globally, the use of modern contraception rose slightly, from 54% in 1990 to 57.4% in 2014. But contraceptive implants need and utilization is exceedingly increasing [3, 4].

Contraceptive discontinuation is common in developing countries (18–63%), and the majority of these discontinuations are among women who are still in need of contraception [5]. According South African Family Practice Implanon discontinue was up to 43% of women prior to completion of the 3 years, most of whom do so because of irregular bleeding patterns and other related reasons. Implanon does have an effect on bleeding patterns, with 20% of women experiencing amenorrhea and 50% of women having unpredictable or prolonged bleeding [6]. As a solution to this problem counseling women on expected bleeding patterns has been shown to improve continuation rates for injectable and implantable progesterone contraceptives [7].

Contraceptive use in Ethiopia has shown tremendous increase in the last decade, following the launching of the health extension program (HEP). Although nearly one-third of women still prefer to delay their next birth for at least 2 years, only 8% of women are currently using long acting reversible contraceptive (LARC) methods [8]. To address the gap, since 2009, the government of Ethiopia has embarked on an Implanon scale-up initiative aimed at expanding access to and enhanced use of Implanon at the community level through enabling health extension workers to provide Implanon insertion services [8, 9]. However unintended pregnancies and its consequences are becoming increasingly concentrated among socioeconomically underprivileged women [10, 11].

Although there are studies in long acting reversible contraceptives in Ethiopia, studies on Implanon removal are limited, even published data on the discontinuation rates of Implanon are scarce. Therefore this study will be helpful to assess the community and to determine the current practice in Implanon discontinuation. Hence findings from this study will be helpful to determine the discontinuation rates and factors associated to early discontinuation rate of Implanon in the community.

## Main text

### Method and material

Institution based cross sectional study design was conducted. The study population was made of all women who requested for Implanon removal during the study period at the selected Mekelle health institutions.

### Sample size determination

The sample size for this particular study was calculated using the formula for a single population proportion considering the following assumptions: A 95% confidence level, a margin of error (0.05), a 16% proportion of early Implanon discontinuation rate was taken from a study conducted in Tigray Ofela Woreda [12].

$${\text{n}} = \frac{{\left( {{\text{Z}}\upalpha/ 2} \right)^{ 2} {\text{p}}\left( { 1- {\text{p}}} \right){\text{n}}}}{{{\text{d}}^{ 2} }}\quad {\text{n}} = \frac{{\left( { 1. 9 6} \right)^{ 2} \left( {0. 1 6} \right) \, \left( {0. 8 4} \right)}}{{\left( {0.0 5} \right)^{ 2} }} = 208$$

Since the topic was sensitive we adding a 10% sample for non-response rate, and the total sample size were 229.

### Sampling technique and procedure

There are 14 health facilities in Mekelle city. Of those seven were selected by simple random sampling. Then samples were proportionally allocated to the selected health facilities based on cases flow rate. Finally, among all women who request to remove Implanon in the selected health institution, samples were selected using Systematic random sampling technique. Assuming K as 2, participants was selected every second case. The first eligible women were selected using the lottery method.

### Operational definitions

- Early Implanon discontinuation: removal of Implanon before completion of 2.5 years of Implanon after insertion [12].

### Data collection tool and procedure

A semi-structured and pre-tested interview-based questionnaire with both open-ended and closed-ended questions was used to collect the data. The questionnaire comprises socio demographic characteristics, reproductive and obstetric history, knowledge and utilization past contraceptive history, counseling status, the role of partner and reasons to remove Implanon types of questions. The questionnaire was adopted from different studies [13, 14].

### Data analysis procedure

The data were checked for completeness, inconsistencies, then coded, entered, cleaned and analyzed in Epi-Info version 7 and Statistical Package for Social Sciences (SPSS version 22). The data are presented with texts, tables, and figures. The binary logistic regression model was used to test the association between dependent (early Implanon discontinuation) and independent variables (Socio demographic, side effect, pre-insertion counseling, future pregnancy desire, partner influence, follow up and service satisfaction). Goodness-of-fit test was cheeked through Hosmers and lemeshow (P = 0.33). Variables multicollinearity were tested and VIF was (< 10) and tolerance test (> 0.1). All variables with P value < 0.2 in bivariate analyses were included in the multivariable analysis. The strength of association was measured by using an adjusted odds ratio at 95% confidence interval and statistical significance was declared at P-value < 0.05.

### Ethical consideration

The study was conducted after getting ethical clearance from Mekelle University, College of Health Science Institutional Review Board (ERC0912/2017). A written informed consent was obtained from participants whose age was above than 18 and an assent form legal guardian or parent and additional consent were obtained from those less than 18 years old participants. Participants were anonymous and the information provided by each respondent was kept confidential.

### Results

#### Socio demographic characteristic

Unexpectedly, all respondents able to complete questionnaires and the response rate was 100%. The median age of respondents’ was 26 ± 9 IQR years ranging from 16 to 51 years. Most mothers with an age of 30 and above were good Implanon users (72.5%) compared to whose age was 15 up to 19 (51.4%). The largest ethnic group was Tigray 210 (91.7%) followed by Amhara 16 (7%). Regarding the educational status of the participants, fifty-seven (24.9%) mothers were illiterate and fifty-six (24.5%) had more than secondary educational level and the rest 116 (50.6%) attend elementary and secondary school. Mothers with a higher educational status showed an improved Implanon use (73%) than illiterate mothers (49.1%) Participant’s occupational status showed that, 118 (51.1%) were a housewife and the rest 55 (24%), 44 (19.2%) and 13 (5.7%) were civil servant, privet business owner and students respectively. The median monthly income of participants was 2000 with IQR + 1370 (Table 1).

**Table 1:** Socio demographic characteristics of respondent in Mekelle city (N = 229) northern Ethiopia, 2017

Variables	Number (%)	Discontinuation	
		Early N (%)	Late N (%)
Age			
15–19	37 (16.2)	18 (48.6)	19 (51.4)
20–24	48 (21)	21 (43.8)	27 (56.3)
25–29	68 (29.7)	25 (36.8)	43 (63.2)
30–34	40 (17.5)	11 (27.5)	29 (72.5)
> 35	36 (15.7)	12 (33.3)	24 (66.7)
Marital status			
Married	166 (72.5)	58 (34.9)	108 (65.1)
Single	41 (17.9)	20 (48.8)	21 (51.2)
Divorced	22 (9.6)	9 (40.9)	13 (59.1)
Religion			
Orthodox	202 (88.3)	80 (39.6)	122 (60.4)
Muslim	23 (10)	5 (21.7)	18 (78.3)
Protestant	4 (1.7)	2 (50)	2 (50)
Occupation			
House wife	118 (51.1)	45 (38.5)	72 (61.5)
Civil servant	55 (24)	21 (38.2)	34 (61.8)
Merchant	44 (19.2)	15 (34.1)	29 (65.9)
Student	12 (5.7)	6 (46.2)	7 (53.8)
Ethnicity			
Tigray	210 (91.7)	80 (38.1)	130 (61.9)
Amhara	16 (7)	5 (31.3)	11 (68.8)
Afar	3 (1.3)	2 (66.7)	1 (33.3)
Level of education			
Cannot read and write	57 (24.9)	29 (50.9)	28 (49.1)
Elementary	50 (21.8)	20 (40)	30 (60)
Secondary	66 (28.8)	23 (34.8)	43 (65.2)
More than secondary	56 (24.5)	15 (26.8)	41 (73.2)

#### Early Implanon discontinuation rate and reason

**Fig. 1:**
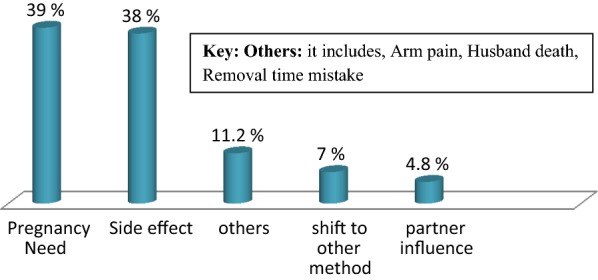
Reasons for early implanon discontinuation among respondents in Mekelle city (N = 229) Northern Ethiopia 2017

Among those 229 mothers who requested to remove Implanon at the study period, 87 mothers remove the Implanon before two and halve year and the early Implanon discontinuation rate was 38%, 95% CI (32–44%). The minimum requested duration was 3 month. Generally, among those who requested removal, the distribution shows that, discontinuation rate was (2.6%) within 6 months, (15.7%) 1 year, (19.7%) in 2 years and (62%) in 3 years. Based on the participants report on their reason for their discontinuation, ninety (39.3%) need to have child, 87 (38%) raised side effect, 16 (7%) shift to another method and 11 (4.8%) reasoned partner influence and rest 25 (11.2%) remove due to different reasons, such as arm pain, husband death and used for 3 years (Fig. 1).

#### Factors associated with early Implanon discontinuation

Socio demographic, obstetric, contraceptive history and counseling related variables was assessed for association with early Implanon removal. In Binary logistic regression, number of child, counseling on Implanon, service satisfaction, Implanon side effect, and appointment after Implanon insertion were significantly associated with Implanon discontinuation (P-value < 0.05). Level of education and Implanon insertion place were also introduced into the model as the P-value was < 0.2. However, in multivariable logistic regression Those mother who were illiterate were more likely to remove Implanon early as compared to those whose educational level was secondary [AOR: 95% CI 0.35 (0.14, 0.82)] and who had more than secondary education [AOR: 95% CI 0.23 (0.09, 0.59)]. Those mothers who was not counseled about the side effect at insertion time were 2.45 times high to remove the Implanon early as compared to those who were counseled (AOR: 95% CI 2.45 (1.05, 5.69) (Table 2).

**Table 2:** Factors associated with early implanon discontinuation rate among respondents (N = 229) in Mekelle Northern Ethiopia 2017

Variable	Discontinuation rate		COR (95% CI)	AOR (95% CI)	P-value
	Early [n (%)]	Late [n (%)]			
Level of education					0.015
Cannot read and write	29 (50.9)	28 (49.1)	1	1	
Elementary	20 (40)	30 (60)	0.64 (0.29, 1.38)	0.50 (0.20, 1.20)	
Secondary	23 (34.8)	43 (65.2)	0.51 (0.25, 1.06)	0.35 (0.14, 0.82)	
More than secondary	15 (26.8)	41 (73.2)	0.35 (0.16, 0.77)	0.23 (0.09, 0.59)	
Have child before insertion					0.57
Yes	47 (31.3)	103 (68.7)	0.44 (0.25, 0.78)	0.52 (0.34, 1.01)	
No	40 (50.6)	39 (49.4)	1	1	
Counseled before insertion					0.036
Yes	66 (34)	128 (66)	1	1	
No	21 (60)	14 (40)	2.90 (1.39, 6.08)	2.45 (1.05, 5.69)	
Implanon insertion place					0.43
Health center	56 (39.4)	86 (60.6)	2.82 (0.76, 10)	2.48 (0.56, 10.1)	
Hospital	11 (50)	11 (50)	4.3 (0.9, 19)	4.10 (0.75, 22)	
Meristops	17 (34.7)	32 (65.3)	2.3 (0.5, 9.20)	2.34 (0.47, 11)	
Family guidance	3 (18.8)	13 (81.3)	1	1	
Service satisfaction					0.101
Yes	12 (6)	194 (94)	1	1	
No	6 (26)	17 (74)	5.7 (2.47, 7.13)	2.57 (0.93, 5.72)	
Implanon side effect					0.012
Yes	73 (43.5)	95 (56.5)	2.58 (1.3, 5.04)	2.66 (1.23, 5.72)	
No	14 (23)	47 (77)	1	1	
Appointment after insertion					0.088
Yes	45 (32.2)	101 (67.8)	0.43 (0.25, 0.75)	0.53 (0.26, 1.09)	
No	42 (48.8)	41 (51.3)	1	1	

The odds of participants who had Implanon side effect increased by 2.66 times to remove early as compared to those who didn’t have any side effect. (AOR: 95% CI 2.66 (1.23, 5.72). Unexpectedly in this study service satisfaction didn’t show an associate in multivariate analyses with early Implanon removal [AOR = 2.57 95% CI (0.93, 5.72)] (Table 2).

### Discussion

In this study the overall early Implanon discontinuation rate was 38% 95% CI (32–44%). This result is higher than the data from Tigray Ofla (16%) [12] this might be due to the denominator difference; the previous study uses mothers who inserted the method within 1 year to the survey but in this study, the denominators were a mother who was in need of removal. The result is also higher than others studies in Nigeria (26.1%) [15] but lower than the study conducted in Debre Markos (46.5%), [13] and Sudan (43.5%) [16]. This discrepancy might be due to sample size, time, socio-cultural difference, and governmental implementations done in minimizing early removal of Implanon.

This study showed that discontinuation rate was (2.6%) within 6 month, (15.7%) 1 year, (19.7%) 2 year and (62%) in 3 years. This result is higher than the study done in (Engu) Nigeria [17]. in that study the removal rate was (3%) within 6 months, (8.1%) in 1 year and (19.3%) removed in 2 year but our study result was lower than the study done in Debre Markos which documents 10.5%, 23.9%, 38.2% and 46.5% removal at 6, 12, 24 and 36 months respectively [13]. The reason for this discrepancy might be the counseling service given during inception of the mothers into the method and continuous follow up on those mothers. For instance, in this study, 84.7% of the participants were counseled on the side effect and effectiveness of the contraceptive method, whereas in the study done in Debre Markos 79% of the participants were counseled [13]. Other factors such as study denominators, sample size, and the socio-cultural difference might be a reason for this discrepancy.

In this study educational status was reviled as a predictor to early removal of Implanon. Illiterate mothers were more likely to remove Implanon early as compared to those whose educational level was secondary [AOR: 95% CI 0.35 (0.14, 0.82 P 0.015)] and more than secondary education [AOR: 95% CI 0.23 (0.09, 0.59 P 0.016)]. This result is in contrary with the previous study done in Debre Markos which shows discontinuation was higher in those mothers who attend more than secondary school those who had no formal education (AOR = 2.2, 95% CI 1.16–4.16) [13]. But this result was supported by study done in Durame town which shows continuation rate in literate mother were fourfold higher than their counterpart illiterate mothers (AOR 4.09, 95% CI 1.68, 9.58) [18] and study done in Ilorin, Nigeria [15] which strongly agreed that accepting and continuation of Implanon was influenced by the educational attainment. This might be due to the reason that those mothers with higher educational level can understand the possible side effect of the method and able to coupe the method by comparing this with the benefit. Another reason might be literate mothers can access information from different sources about the method other than health caregiver which helps them to realize the continuation beside the different reasons which can lead them to discontinue the method early.

The other predictor that showed significant association with early removal of implant was counseling during the insertion. This study showed mothers who were not counseled about the effectiveness and side effect at insertion time were 2.5 times high to remove the Implanon early as compared to those counseled [AOR = 95% CI 2.45 (1.05, 5.69, P 0.036)]. This result is in line with study done in Debre Markos [AOR = 1.2, CI (1.02–3.89, P 0.044)] [13] Nigeria [17]. The most cited reason for this effect were, effective counseling at insertion time on possible side effect of the method will help the mother to accept the possible side effects and minimize removal of the method at an early time. In addition to this use of appropriate counseling type and timing might also help the women to cope with minor side effects and strengthen the continuation of the method.

Similar to other previously done studies this study also shows that mothers who experienced Implanon side effect were by 2.66 times more likely to remove the methods early as compared to those who didn’t experience a side effect [AOR = 95% CI 2.66 (1.23, 5.72, P 0.012)]. This result was in line but smaller than the study done in Debre Markos [AOR = 3.14, CI (1.7–5.54, P 0.001)] [13] another study done in Oflawereda 2.79 (1.10–7.07) [12] and study done in Kenya [AOR 3.582 (1.637–7.840)] [16]. This result was also supported by other studies from Butajira [19] United Kingdom [20] and Nigeria which shows that side effect was a reason for significant numbers (12–91%) of early removal of the method. This might be due to the fact that, side effect directly affects mother tolerance and use which precipitates them to change the method or withdraw from and Implanon use. On the other hand, family planning provider’s gap on skill and knowledge concerning the management of side effects could raise the discontinuation habit of those mothers in need of removal.

## Conclusion

The study revealed that early Implanon discontinuation was high and the main reason for their early removal was women pregnancy need and Implanon side effects. The study also suggests that lower educational status of mothers increase the likelihood to remove the method early but effective counseling on side effect and effectiveness of the method during insertion decrease early Implanon discontinuation.

## Limitation

- A woman may have difficulty of remembering when she inserts Implanon mean that recall bias.
- The study was conducted in an urban health institution setting, though the majority of the populations live in rural setting.
- Use of only quantitative method was also another limitation of the study.

## Abbreviations

CPR: contraceptive prevalence rate
EDHS: Ethiopian Demographic and Health Survey
FMOH: Federal Ministry of Health of Ethiopia
HEP: health extension program
HIV: human immune deficiency virus
LARC: long acting reversible contraceptive
